# Exploiting Botulinum Neurotoxins for the Study of Brain Physiology and Pathology

**DOI:** 10.3390/toxins10050175

**Published:** 2018-04-25

**Authors:** Matteo Caleo, Laura Restani

**Affiliations:** CNR Neuroscience Institute, via G. Moruzzi 1, 56124 Pisa, Italy; caleo@in.cnr.it

**Keywords:** synaptic transmission, SNAP-25, epilepsy, Parkinson’s disease, neurotransmission blockade, electrical activity, prion disease

## Abstract

Botulinum neurotoxins are metalloproteases that specifically cleave *N*-ethylmaleimide-sensitive factor attachment protein receptor (SNARE) proteins in synaptic terminals, resulting in a potent inhibition of vesicle fusion and transmitter release. The family comprises different serotypes (BoNT/A to BoNT/G). The natural target of these toxins is represented by the neuromuscular junction, where BoNTs block acetylcholine release. In this review, we describe the actions of botulinum toxins after direct delivery to the central nervous system (CNS), where BoNTs block exocytosis of several transmitters, with near-complete silencing of neural networks. The use of clostridial neurotoxins in the CNS has allowed us to investigate specifically the role of synaptic activity in different physiological and pathological processes. The silencing properties of BoNTs can be exploited for therapeutic purposes, for example to counteract pathological hyperactivity and seizures in epileptogenic brain foci, or to investigate the role of activity in degenerative diseases like prion disease. Altogether, clostridial neurotoxins and their derivatives hold promise as powerful tools for both the basic understanding of brain function and the dissection and treatment of activity-dependent pathogenic pathways.

## 1. Introduction

Botulinum neurotoxins (BoNTs) are the pathogenic agents responsible for the manifestation of botulism. The typical flaccid paralysis of botulism induced by BoNTs is due to blockade of cholinergic neurotransmission at the neuromuscular junction and autonomic terminals [1,2,3].

These toxins are produce by anaerobic bacteria of the genus Clostridium and are among the most potent naturally-occurring substances. The family of BoNTs comprises seven antigenically distinct botulinum neurotoxins (BoNT/A–BoNT/G). For serotypes A, B, E, and F, several subtypes have been described based on differences in amino-acid sequences. For BoNT/A, at least eight subtypes (named A1 to A8) are currently known with different enzymatic activity and toxicological properties [4,5,6].

BoNTs share a common molecular structure and are composed of a disulphide-linked, ~100-kDa heavy chain and ~50-kDa light chain. They are metalloproteases that bind to presynaptic terminals, enter the cytosol and block neurotransmitter release by specific cleavage of proteins of the soluble *N*-ethylmaleimide-sensitive factor attachment protein receptor (SNARE) complex. The SNARE complex is necessary for synaptic vesicles fusion, thus the net effect is blockade of neurotransmitter release [3,7,8]. The target protein differs according to BoNTs serotype. BoNT/A and E cleave synaptosomal associated protein of 25 kDa (SNAP-25); BoNT/C acts on both SNAP-25 and syntaxin; BoNT/B, D, F and G cleave vesicle-associated membrane proteins (VAMPs, also known as synaptobrevins).

Despite their toxicity, they produce a prolonged but reversible action at the synapses. Thus, it has been speculated, already decades ago, that small amount of BoNTs could be used therapeutically to treat disorders characterized by hyperexcitability. Historically, the first to make therapeutic use of BoNT/s was Alan B. Scott in the 1970s, for the treatment of strabismus [9]. Subsequently, the Food and Drug Administration has continuously increased the approved uses for botulinum neurotoxin A1 (BoNT/A1). BoNT/A1 is indeed the most used serotype in clinical practice, because the protease has a persistent activity and this allows long lasting duration of the therapeutic effects (months).

To date, approved indications include focal dystonias, spasticity, cosmetic treatments and migraine, and several other applications are emerging. In all of these cases, minute amounts of BoNT are administered in peripheral muscles to locally inhibit transmitter release.

However, BoNTs are also effective in blocking transmitter release at central synapses when directly delivered into the brain [10].

Here we will review literature data reporting BoNTs effects following direct injection into the central nervous system. Specifically, we will describe how these potent and selective synaptic blockers may be exploited to gain insight into mechanisms of brain physiology and dysfunction.

## 2. Action of BoNTs on Central Synaptic Terminals

BoNTs enter central neurons mainly via activity-dependent synaptic endocytosis, indeed depolarization increases toxins uptake [11,12,13,14]. At least for BoNT/A, neuronal entry also occurs via an alternative pathway independent of synaptic vesicle endocytosis [15,16], which may direct the toxin to the retroaxonal transport pathway [17,18].

Analyses on brain synaptosomes have demonstrated that BoNTs (mainly studies on BoNT/A) interfere with neurotransmitter release of acetylcholine, glutamate, noradrenaline, serotonin and dopamine from central synases ([10]). It is interesting to note that GABAergic terminals are more resistant to BoNT/A intoxication compared to excitatory (glutamatergic) terminals [19,20]. One reason could be that SNAP-25, the synaptic target of BoNT/A, is less expressed in inhibitory than in glutamatergic terminals [20,21]. For example, SNAP-25 is almost absent in perisomatic inhibitory terminals impinging onto principal neurons in the pyramidal layer of hippocampal CA1 [22]. However, recent electrophysiological recordings in embryonic stem cell-derived neurons (ESNs), showed that miniature Inhibitory Post Synaptic Currents (mIPSC) frequencies were already reduced more than 70% 30 min after BoNT/A intoxication, while decrease in miniature Excitatory Post Synaptic Currents (mEPSC) frequencies was detectable only after 70 min [23]. This finding supports the initial increase in frequency of mPSCs in the first hour after BoNT/A treatment, followed by basically a complete silencing of activity around 15 h [23].

Silencing of spontaneous and evoked excitatory postsynaptic potentials was already demonstrated in hippocampal neurons [24,25]. Accordingly, in vivo delivery of BoNT/A or BoNT/E in rodent hippocampus prevents neuronal spiking activity in hippocampal CA1 [26,27].

It is worth noting that BoNT/A produces an efficient blockade of neurotransmitter release by cleaving a small percentage (about 10%) of the SNAP-25. This seems to be due to the dominant negative effect of BoNT/A-truncated SNAP-25 [28]. However, it possible to rescue BoNT/A-induced blockade of neurotransmission by increasing extracellular calcium concentration. Although BoNT/A and BoNT/E share the same synaptic target (SNAP-25), this rescue with calcium is not possible with BoNT/E, probably because serotype E cleaves a larger fragment at the C-terminus of SNAP-25 [24,29].

At the ultrastructural level, our group investigated the morphological changes induced by local delivery of BoNT/A into the hippocampus [30,31]. Hippocampal samples were analyzed at different times following BoNT/A injection (2, 4, 8 weeks). Observation of electron microscope images, focused on the CA1 stratum radiatum, revealed that BoNT/A induced an accumulation of synaptic vesicles. This accumulation triggered an enlargement of presynaptic terminals which was maximal at 4 weeks [30]. It is noteworthy that these changes were detectable basically only in asymmetric, excitatory synapses, and not in symmetric, GABAergic synapses, confirming a preferential effect of BoNT/A on excitatory terminals [20,21,22,30]. Axonal enlargements were also observed within the striatum injected with BoNT/A. These enlargements result positive for choline acetyltransferase (ChAT) and tyrosine hydroxylase (TH) in rats, but positive only for ChAT in mice [32,33].

## 3. BoNTs for the Study of Brain Physiology

A typical feature of BoNTs is that their action is prolonged but reversible. These characteristics make BoNTs, in particular BoNT/E which produces a short-lived blockade, ideal tools to study brain physiology. BoNTs allow a transient “silencing” of specific brain regions after a single administration, which is experimentally more convenient compared to other drugs that need to be continuously infused (e.g., tetrodotoxin or muscimol) [34].

Luvisetto, Pavone and collaborators were among the first to test the impact of direct brain injections of BoNTs in mice. They performed intracerebroventricular (icv) injections of sub-lethal doses of BoNT/A or BoNT/B and assessed various behavioral responses [35], such as active avoidance and object recognition. They also analyzed BoNTs effects on pharmacologically induced locomotor activity. The results indicated no effect on active avoidance acquisition, while there were impairments in the novel object recognition task, and amplified effects of drugs which induce locomotor activity [35]. The same group also tested the effects of central administration of BoNT/A on pain mechanisms [36]. They used a mouse model of formalin-induced pain (injection of formalin into the hindpaw) and the licking response as an index of pain. The data showed that intracerebral BoNT/A affected the licking response in the second phase of formalin test, similar to the effects obtained with peripheral administration [36,37]. Anti-nociceptive effects of central administrations of BoNT/A were later confirmed by other groups in various models of pain [38,39].

Our group has exploited BoNT/E to obtain a sustained but reversible blockade of neurotransmission for about 2 weeks in specific brain regions [27,40]. In particular, to investigate the role of cortical activity in the maturation of visual function, we unilaterally injected BoNT/E into the visual cortex (V1) in rat pups, at the time of eye opening [40]. BoNT/E injection produced a unilateral silencing of V1 for about 2 weeks, completely abolishing visual responses during the so called “critical period” for development of cortical function [41]. We performed electrophysiological recordings 3 weeks following BoNT/E injection (when cleaved SNAP-25 was no longer detectable), in order to assess visual system development when electrical activity was recovered, i.e., at the completion of the normal critical period. We found that BoNT/E-induced silencing of cortical activity did not allow normal maturation of visual function, keeping visual acuity low and extending the duration of the critical period [40]. We also evaluated if these deficits were persistent, or if they reflected only a delay in visual function maturation. Thus we performed behavioral and electrophysiological analyses at a longer time point (more than 2 months following toxin injection), and we confirmed a persistent impairment in visual performance. In conclusion, exploiting BoNT/E delivery to induce a transient silencing of cortical activity during the critical period allowed us to demonstrate that intrinsic cortical activity is necessary for a correct development of visual function [40].

Long-lasting serotypes such as BoNT/A and BoNT/B could be useful to create animal models of pathologies (e.g., dementia, [42]) or to treat hyperexcitability [26] (see below). However, these models could also offer basic knowledge about the role of specific brain regions in behavioral performance. For example, BoNT/B injection into the entorhinal cortex in adult rats produce learning and memory impairments as assessed by maze tests [42].

Similarly, BoNT/E hippocampal injection in adult rats induces deficits in spatial learning during the Morris water maze task, but since BoNT/E action is short-lived, the impairments are completely reversible and confirm a key role of hippocampus in spatial learning [26].

Mapping of the spread of BoNT/E via immunostaining for intact and cleaved SNAP-25 [40,43] demonstrates that toxin action remains confined to the cortical areas close to the injection site, thus allowing regional specificity of the synaptic blockade. Toxin diffusion can be further limited via the use of convection-enhanced delivery (CED), which provides a more homogeneous distribution than conventional bolus injection and does not damage the surrounding tissue [44,45,46,47].

## 4. Exploiting BoNTs in Pathological Brain Conditions

We have already reported examples of how BoNTs could be exploited to study the role of electrical activity in physiological, developmental brain processes [40]. In addition, BoNTs delivery could be useful to address the impact of electrical activity in neurodegenerative pathologies. Indeed, while it is known that synaptic degeneration precedes cell loss (e.g., [48]), little is known about mechanisms that tag synapses for degeneration. In this context, our group hypothesized that in a hippocampal mouse model of prion disease (a neurodegenerative disease associated with aggregates of misfolded proteins), synaptic degeneration was activity-dependent. To verify this hypothesis, we injected BoNT/A into the hippocampus of mice with prion disease and we analyzed synaptic degeneration at the ultrastructural level by electron microscopy [30]. Contrary to our expectations, we failed to find differences in the density of degenerating synapses between BoNT/A- and vehicle-injected prion mice. The morphology of the degenerating synapses was also indistinguishable between the two groups. These experiments challenge the idea that dysfunctions in synaptic vesicle release trigger the elimination of synaptic boutons, at least in prion-induced neurodegeneration [30,31].

Recently, Spalletti et al. (2017) used BoNT/E to produce a transient silencing of the contralesional hemisphere in a mouse model of focal stroke in the motor cortex. One of the main hypothesis in the stroke field is the “inter-hemispheric competition model”, which posits an enhanced transcallosal inhibition from the healthy to the lesioned side. To reduce this interhemispheric inhibition, the authors applied BoNT/E to block activity in the contralesional motor cortex immediately after the stroke. They found a significant recovery of motor function in the treated animals. Importantly, functional recovery was further enhanced when the silencing of the healthy side was coupled with physical rehabilitation of the affected arm [43].

Since BoNTs block neurotransmitter release, it is not unexpected that they have been exploited to treat, similarly to the peripheral nervous system, pathologies characterized by hyperexcitability. The most frequent category of central pathologies associated with hyperexcitability is epilepsy. About six millions of persons in Europe develop epilepsy, and around 30% of these are pharmaco-resistant [49]. This means that there are no drugs available to suppress or decrease their seizures. In the worst cases, the only clinical solution is a surgical intervention which physically removes the main epileptic focus. Consequently, efforts for discovering new therapeutic treatments are warranted. Our group and others have investigated whether central, local BoNTs delivery could suppress seizures in animal models of epilepsy [4,26,27,45,50]. The first serotype used was BoNT/E, tested in animal models of acute seizures, triggered by hippocampal administration of pro-convulsant agent kainic acid (KA) [26]. To measure BoNT/E effects, authors performed behavioral and electrographic analyses, demonstrating that BoNT/E delivery is effective in decreasing number and duration of seizures triggered by KA. BoNT/E effects were not limited to the electrophysiological level, but the toxin had an impact also on hippocampal histopathological changes, such as neuronal loss. This neuroprotection likely depends on blockade of excitoxicity phenomena occurring during prolonged electrical activity [26]. The neuroprotective action elicited by BoNT/E has been demonstrated also in a model of focal ischemia [51]. The potent vaso-constricting peptide endothelin-1 (ET-1) was delivered intrahippocampally in adult rats, followed 20 min later by BoNT/E injection in CA1. To evaluate BoNT/E action on excitoxicity, that is, on glutamate release, the authors performed in vivo microdialysis. Data showed that BoNT/E-injected rats had a decreased glutamate release. This synaptic effect was matched with a decrease in CA1 neuronal loss, as measured by immunohistochemistry [51]. Thus, the neuroprotective action by BoNT/E depends on the inhibition of the release of glutamate and occurs via downregulation of proapoptotic proteins, such as caspase-3 [52].

Based on these initial, encouraging data on acute seizures, BoNT/E was tested also in a mouse model of chronic seizures that resembles mesial temporal lobe epilepsy (MTLE), one of the most common pharmacoresistant forms of epilepsy in humans, obtained by intrahippocampal injection of KA [27,50]. The authors initially tested the impact of BoNT/E delivery on epileptogenesis (i.e., the development of spontaneous ictal events) following an episode of status epilepticus triggered by KA. The findings indicated that BoNT/E-mediated synaptic blockade during epileptogenesis was not effective in blocking the occurrence of spontaneous seizures. However, BoNT/E treatment was associated with histopatological protection; there was less neuronal loss in CA1 and the dispersion of granule cells in the dentate gyrus was potently prevented [27]. In a second work, the authors investigated if BoNT/E delivery was sufficient to reduce seizures during the chronic phase of epilepsy [50]. Mice injected with KA were implanted with bipolar electrodes, and after a period of baseline recording sessions, BoNT/E was infused directly into the epileptic hippocampus. Subsequent electrophysiological recordings clearly proved that BoNT/E delivery produces a reduction in total seizure duration and frequency [50].

One may argue that to be practically useful in the treatment of epilepsy, focal treatments require a long duration of action. Other serotypes of BoNTs with a prolonged proteolytic activity, like BoNT/A or BoNT/B, are ideal tools. Indeed, a couple of studies have used these serotypes to block seizures for longer periods in the amygdala kindling model, an experimental paradigm that allows to follow seizures for weeks to months. Gasior and colleagues (2013) directly infused BoNT/A or BoNT/B into the amygdala, via convection-enhanced delivery (CED) [45]. Therapeutic effects of both toxins were assessed by measuring after-discharge threshold and other parameters of the amygdala-kindled seizures at different times (3, 7, 10, 15, 21, 35, 50, and 64 days) after the administration. Results pointed to the anti-convulsant effects of both toxins, as assessed with EEG measures (i.e., elevation in after-discharge threshold of stimulation and seizures duration). The anti-convulsant action persisted until day 50. It interesting to note that, whilst BoNT/B was also effective in reduction of behavioral seizures, BoNT/A did not reach significance values in this parameter [45].

Another manuscript exploited infusion of BoNT/A (specifically serotype A2) to reduce seizures in kindled mice [53]. In half of the animals, BoNT/A2 was able to completely block the appearance of seizures. In addition, the toxin decreases the level of seizures, at least until 18 days following injection.

Taken together, these results suggest that BoNTs are quite effective in amelioration of epileptic activity, and they could be potentially used as focal antiepileptic treatments.

One might envision another possible “diagnostic” use of BoNTs in epilepsy, especially for BoNT/E, which has the shorter duration of action. In patients eligible for resection surgery, it is fundamental to precisely map brain epileptic foci, to remove all the hyperexcitable areas and render the patient seizure-free after surgery. The mapping is usually performed by non-invasive imaging techniques (such as magnetoencephalography (MEG) and functional MRI (fMRI)), or by EEG with chronically implanted electrodes [54], however the results are not always satisfactory, and patient could suffer of residual seizures also after surgery. In this context, local delivery of botulinum toxins could represent a strategy to functionally map the epileptogenic areas, and check whether the silencing of the presumptive focus is effective in abolishing seizures.

Another promising application of local delivery of BoNT/A is the therapeutic treatment of movement disorders and neurotransmission dysfunction typical of Parkinson’s disease (PD). PD is characterized by an imbalanced cholinergic hyperactivity in the striatum, due to the loss of dopaminergic neurons of the substantia nigra. Since BoNT/A blocks neurotransmitter release, including acetylcholine (ACh), the toxin was injected directly into the striatum, in animal models of PD [32,33,55,56,57]. In particular, the rodent model of 6-hydroxydopamine (6-OHDA) produces a hemi-parkinsonism. Wree and colleagues (2011) tested effects of BoNT/A injected 6 weeks following lesion with 6-OHDA. BoNT/A action was evaluated using the apomorphine-induced contralateral rotation test. Apomorphine is a dopamine (DA) receptor agonist and stimulates the supersensitive dopamine receptor D2 (DRD2) in the lesioned hemisphere, causing a net rotation away from the side of the lesion, that is, anti-clockwise [58]. Infusion of BoNT/A into the ipsilateral, lesioned striatum is able to reverse this rotation movement until 3 months [32]. Authors observed also enlarged axonal varicosities in BoNT/A (BiVs) injected-animals (possibly due to synaptic vesicles accumulation as seen in hippocampus by Caleo and co-authors [30]). Immunohistochemical analysis revealed that these axonal varicosities were cholinergic, but some of the BiVs were found to be positive for tyrosine hydroxylase (TH) [32,55]. In a subsequent work, these cholinergic varicosities induced by BoNT/A were investigated in detail [55]. They evaluated the number of ChAT-positive interneurons as well as the density and the volumetric size of the BiVs. In the ipsilateral side of BoNT/A-injected rats, with 6-OHDA lesion, the numeric density of BiVs reached a maximum 3 months after BoNT/A, while their volume increased during the whole time course of the experiment. However, no differences were detectable in the number of ChAT-positive neurons, up to 1 year following BoNT/A injection. This last result is important because it speaks in favor of a lack of cytotoxic effects of BoNT/A [55].

A similar study has been performed in mice, to extend possible therapeutic BoNT/A applications to genetics mouse models of PD [33]. Authors injected increasing doses of BoNT/A, finding no differences in the number of ChAT-positive interneurons. Increasing BoNT/A doses (from 25 pg to 200 pg), led to an increased BiV volume, and a decreased number of small BiVs. It is noteworthy that, in contrast to rats, TH-immunoreactive BiVs were not found in BoNT/A-infused mice [33].

Intrastriatally injected BoNT/A appears also to induce changes in receptor expression, likely due to activity silencing. For example, BoNT/A reduced density of dopamine receptor D2/D3, whereas other key receptors (such as dopamine 1 (D1), noradrenergic (a1 and a2) and serotonergic (5HT2A) receptors) remained basically unaltered in rats [57]. Since authors found few weeks after unilateral 6-hydroxydopamine (6-OHDA) lesion a significant increase of D2/D3 receptor ratio, the therapeutic effects of BoNT/A probably resides in reducing the interhemispheric imbalance in D2/D3 receptor density in lesioned rats.

Altogether, these results indicate how intracerebrally injected BoNTs could induce synaptic silencing and long-lasting changes in neurotransmitter-related proteins, that ultimately produce therapeutic benefits (see Table 1 for a summary).

## 5. Intracerebral BoNTs: Future Directions

BoNT clinical indications are continuously increasing, thanks to advantages such as very long duration, high potency, and complete reversibility of action [3].

There is currently considerable interest in developing novel forms of BoNTs with optimized therapeutic properties and neuronal selectivity (i.e., neuromuscular junction vs. sensory endings), which could offer new treatment opportunities. On one hand, the natural repertoire of BoNTs offers a wide variety of molecules with specific actions in neuronal cells and in vivo mouse models [59]. Second, an engineering approach has been taken to modify the pharmacological properties of native toxins by specific mutations. For example, a mutated BoNT/A1 has been created with faster onset and a shorter duration of action than BoNT/A1 wild type [60], opening the way to design BoNT variants with novel and useful properties.

The group of Bazbek Davletov has quite recently developed a new technology, named “protein-stapling”, by which it is possible to re-assemble chimeric clostridial neurotoxins starting from two separate modules, that is, the light chain/translocation domain and the receptor-binding domain [61,62]. This technology is not only useful to safely produce active toxins, but also allows engineering of toxins. The first engineered toxin was an analogue of the botulinum neurotoxin type A, called BiTox. The structural evaluation of BiTox suggests that the re-assembled BoNT/A could be substantially longer than the native molecule. However, BiTox demonstrated similar efficiency to that of native BoNT/A in proteolytic cleavage of SNAP-25 in vitro and in vivo, and thus in neurotransmitter silencing [61]. Interestingly, and clinically relevant, potency of BiTox at the neuromuscular junction is reduced, probably because of the bigger size of the molecule. Thus, systemic toxicity is reduced in BiTox injected subjects, and this represents a considerable advantage for clinical applications [61].

Engineered neurotoxins could also be exploited to enhance the selectivity for selected neuronal populations, combining the receptor-binding domain with different catalytic chains. For example, the same group has combined BoNT/A protease with the TeNT binding domain, allowing intoxication of different neuron populations compared to the native BoNT/A [62]. This chimera has a nociceptive action at central level, but has no action on motoneurons (as it caused neither flaccid nor spastic paralysis), resulting safer and potentially relevant for medical applications. On the other side, engineered toxins are interesting also for basic neuroscience research. Indeed, this chimera, following direct delivery into the rat visual cortex, was able to modulate sensory function [62].

## Figures and Tables

**Table 1:** Exploiting botulinum neurotoxins (BoNTs) in pathological brain conditions. The table summarizes the main studies that have exploited central delivery of botulinum neurotoxins to treat pathological brain conditions.

Disease	Animal Model	Species	BoNT Serotype	Reported Effects	Reference
Epilepsy	intrahippocampal KA	rat	BoNT/E	decreased number and duration of seizures triggered by KA; decreased neuronal loss	Costantin et al, 2005 [26]
	intrahippocampal KA	rat	BoNT/E	downregulation of caspase 3	Manno et al, 2007 [52]
	intrahippocampal KA	mouse	BoNT/E	decreased neuronal loss and dispersion of granule cells (BoNT/E tested during epileptogenesis)	Antonucci et al, 2008 [27]
	intrahippocampal KA	mouse	BoNT/E	reduction of total seizure duration and frequency (BoNT/E tested during chronic phase)	Antonucci et al, 2009 [50]
	amygdala kindling model	rat	BoNT/A BoNT/B	anti-convulsant effects of both toxins (BoNT/B also at behavioral level)	Gasior et al, 2013 [45]
	amygdala kindling model	mouse	BoNT/A2	decreased seizures (in 50% of animals)	Kato et al, 2013 [53]
Ischemia	endothelin 1	rat	BoNT/E	neuroprotective effect (decrease of glutamate release)	Antonucci et al, 2010 [48]
	phototrombotic stroke	mouse	BoNT/E	synaptic silencing of contralateral hemisphere improved motor recovery	Spalletti et al, 2017 [43]
Parkinson’s disease	6-OHDA model	rat	BoNT/A	abolished pathologic rotational behavior; induced ChAT and TH axonal varicosities	Wree et al, 2011 [32]
	6-OHDA model	rat	BoNT/A	induced ChAT and TH axonal varicosities; no changes in ChAT-positive neurons	Mehlan et al, 2016 [55]
	6-OHDA model	mouse	BoNT/A	induced ChAT axonal varicosities;	Hawlitschka et al, 2017 [33]
	6-OHDA model	rat	BoNT/A	changes in receptor expression (rebalance of D2/D3 receptor density)	Mann et al, 2018 [57]
Prion disease	ME7 prion disease	mouse	BoNT/A	electrical activity does not impact on synaptic degeneration	Caleo et al, 2012 [30]
Pain	formalin-induced pain	mouse	BoNT/A	decreased licking response in the second phase of formalin test	Luvisetto et al, 2006 [36]
